# Clinical importance and impact on the households of oseltamivir-resistant seasonal A/H1N1 influenza virus in healthy children in Italy

**DOI:** 10.1186/1743-422X-7-202

**Published:** 2010-08-26

**Authors:** Susanna Esposito, Claudio Giuseppe Molteni, Cristina Daleno, Antonia Valzano, Emilio Fossali, Liviana Da Dalt, Valerio Cecinati, Eugenia Bruzzese, Raffaella Giacchino, Carlo Giaquinto, Carlotta Galeone, Angie Lackenby, Nicola Principi

**Affiliations:** 1 Department of Maternal and Pediatric Sciences, Università degli Studi di Milano, Fondazione IRCCS Ca' Granda Ospedale Maggiore Policlinico, Milan, Italy; 2 Pediatric Department, University of Padua, Padua, Italy; 3 Department of Biomedicine of Evolutive Age, University of Bari, Bari, Italy; 4 Pediatric Department, Università Federico II, Naples, Italy; 5 Infectious Disease Unit, IRCCS Ospedale Giannina Gaslini, Genoa, Italy; 6 Department of Epidemiology, Istituto di Ricerche Farmacologiche Mario Negri, Milan, Italy; 7 Health Protection Agency, London, UK

## Abstract

A resistance of A/H1N1 influenza viruses to oseltamivir has recently emerged in a number of countries. However, the clinical and socioeconomic importance of this resistance has not been precisely defined. As children have the highest incidence of influenza infection and are at high risk of severe disease, the aim of this study was to evaluate the clinical importance and the impact on the households of oseltamivir-resistant seasonal A/H1N1 influenza virus in an otherwise healthy pediatric population. A total of 4,726 healthy children younger than 15 years with influenza-like illness were tested for influenza viruses by real-time polymerase chain reaction in the winters of 2007-2008 and 2008-2009 in Italy. The influenza A virus-positive samples underwent neuraminidase gene analysis using pyrosequencing to identify mutations H275Y and N294 S in A/H1N1, and E119V, R292K, and N294 S in A/H3N2. Among the A/H1N1 subtypes, the H275Y mutation was found in 2/126 samples taken in 2007-2008 (1.6%) and in all 17 samples (100%; p < 0.0001) taken in 2008-2009. No other mutation was identified in any of the A/H1N1 or A/H3N2 influenza viruses. No significant differences were found in terms of clinical importance or impact on the households between the children with oseltamivir-resistant seasonal A/H1N1 influenza virus and those with the wild-type. The spread of H275Y-mutated A/H1N1 seasonal influenza virus is a common phenomenon and the clinical importance and impact on the households of the mutated virus is similar to that of the wild-type in an otherwise healthy pediatric population.

## Finding

Type A influenza viruses are the major cause of influenza worldwide, and subtype A/H1N1 is the most frequently found in the younger population [1,2]. A resistance of A/H1N1 influenza viruses to oseltamivir (mainly due to the H275Y mutation) has recently emerged in a number of countries [3-6]. Oseltamivir-resistant viruses have been associated with a negative disease course and death in high-risk patients [7-9], but there are few data concerning the otherwise healthy population. As children have the highest incidence of influenza infection and are at high risk of severe disease when infected by wild-type influenza viruses, the aim of this study was to evaluate the clinical importance and impact on the households of oseltamivir-resistant seasonal A/H1N1 influenza virus in an otherwise healthy pediatric population.

This multicentre prospective study was carried out in the winters of 2007-2008 and 2008-2009 (from 1 November to 31 March) in the Emergency Room (ER) of five children's hospitals in Italy (Milan, Padua, Genoa, Naples and Bari). The study protocol was approved by the Institutional Review Board of each participating centre; the written informed consent of a parent or legal guardian was required, and the older children were asked for their assent.

The study enrolled subjects aged less than 15 years and without any underlying chronic severe disease who attended an ER for an influenza-like illness as defined by the Italian Ministry of Health http://www.ministerosalute.it. During winter 2007-2008, enrolment took place on two days per week (Wednesday and Sunday); during winter 2008-2009, it took place every day. In both seasons, the enrolled patients' demographic characteristics and medical history were systematically recorded using standardised written questionnaires as previously described [10,11] and, after a complete physical examination, they were classified into disease groups on the basis of signs and/or symptoms using well-established criteria [12].

A nasopharyngeal sample was collected from all of the children using a pernasal flocked swab, and stored in a tube of UTM-RT (Kit Cat. No. 360c, Copan Italia, Brescia, Italy). Each sample underwent real-time polymerase chain reaction (real-time PCR) in order to identify A and B influenza viruses as previously described [7,13]. Reverse transcription plates were set up for subtyping using a Qiagility robot (Qiagen, West Sussex, UK), and multiplex real-time PCR was performed using a Taqman Fast Universal PCR Master Mix (Applied Biosystems, Warrington, UK), fast cycling conditions, and the following primers-probes on an ABI 7500 Fast instrument (Applied Biosystems, Warrington, UK) (final concentrations in parentheses): A/H1-forward GGAATAGCCCCCCTACAATTG (1 μM); A/H1-reverse AATTCGCATTCTGGGTTTCCTA (1 μM); A/H1 probe NED-CGTTGCC-GGATGGAMGBNFQ (0.05 μM); A/H3-forward CCTTTTTGTTGAACGCA-GCAA (1 μM); A/H3-reverse CGGATGAGGCAACTAGTGACCTA (1 μM); A/H3-probe VIC-CCTACAGC-AACTGTTACCMGBNFQ (0.25 μM); B-forward TCACGAAAAATACGGTGGATTAAA (0.75 μM); B-reverse TTTGGTTCCATTGGCMAGCT (0.75 μM); B-probe 6FAM-CCAATATGGGTGAAAACMGBNFQ (0.3 μM).

The A influenza virus-positive samples underwent neuraminidase gene analysis using pyrosequencing to identify mutations H275Y and N294 S in A/H1N1, and E119V, R292K, and N294 S in A/H3N2. Reverse-transcription (RT) PCR was performed on viral RNA using the Qiagen One-Step RT-PCR Kit with using 0.6 μM primer concentrations. The pyrosequencing was performed as previously described [14]. The primers used for PCR and pyrosequencing were N1 275-294 forward GGAGCCGTGGCTGTACTAAAATA; N1 275-294-reverse BIOTIN-CCACGTTTTGATTAAAAGACACC; N1 275-sequence AGTTGAATGCACCCAAT; N1 294-sequence TGTGTGTATGCAGGGAC; N2 119-forward TTTTATCTGACCAACACCACCATAGAG; N2 119-reverse BIOTIN-CGCTAAGGGGTCCTATCATGTACT; N2 119-sequence ACATCTGGGTGACAAGA; N2 292-294-forward BIOTIN-TTCATTGAGGAGGGGAAAA; N2 292-294-reverse GATCAACGCAATGGCTACTG; N2 292-294-sequence GCCTATTGGAGCCTT.

The children's medical history was re-evaluated 5-7 days after enrolment, and until the resolution of their illness, by means of interviews and clinical examinations conducted by trained investigators using standardised questionnaires that also collected information regarding household illnesses and related morbidities [10,11]. All of the data relating to the study children and their households were verified from medical records.

Continuous data were analysed using a two-sided Student's t-test, after checking data were normally distributed (based on the Shapiro-Wilk statistic) and a two-sided Wilcoxon's rank-sum test otherwise. Categorical data were analysed using the contingency table analysis with the Chi-square or Fisher's test, as appropriate. With a 5% type 1 error rate and a power of 80%, 17 subjects in the group of oseltamivir-resistant virus and 85 in the group of wild-type virus were required to show a difference of 30% in the severity of influenza-related events.

A total of 4,726 children (2,599 males; mean age ± standard deviation [SD], 3.34 ± 3.06 years) were enrolled: 1,170 (24.8%) during winter 2007-2008 and 3,556 (75.2%) during winter 2008-2009. Influenza A viruses were detected in 729 (15.4%) children, 164/1,170 (14.0%) in the first year and 565/3,556 (15.9%) in the second; influenza B viruses were detected in 239 (5.1%), 179/1,170 (15.3%) in 2007-2008 and 60/3,556 (1.7%; p < 0.0001) in 2008-2009. In the two seasons, none of the influenza A or B positive children has been previously vaccinated against influenza.

Influenza A viruses in 150 of the 164 sample collected in the first year (91.5%) and 512 of the 565 samples collected in the second year (90.6%) were subtyped and analysed using pyrosequencing. Subtype A/H1N1 was identified in respectively 126/150 (84%) and 17/512 cases (3.3%; p < 0.0001), and subtype A/H3N2 in 24/150 (16%) and 495/512 (96.7%; p < 0.0001). Among the A/H1N1 subtypes, the H275Y mutation was found in 2/126 samples (1.6%) in 2007-2008 and in all 17 samples (100%; p < 0.0001) in 2008-2009. No difference in resistance frequency was observed between the different centers during both seasons. No other mutation was identified in any of the A/H1N1 or A/H3N2 influenza viruses.

Table 1 shows the demographic and clinical characteristics of the children with A/H1N1 influenza infection, by the presence or absence of the H275Y mutation, and the impact on the households of the infection on their households No statistically significant differences were found in terms of gender, age, viral load, diagnosis at enrolment, clinical outcome, pharmacological treatment or impact on the households between the children with oseltamivir-resistant seasonal A/H1N1 influenza virus and those with the wild-type.

**Table 1:** Demographic, clinical and socioeconomic characteristics of the children with A/H1N1 influenza infection, by the presence or absence of the H275Y mutation.

Characteristics	Oseltamivir-resistant seasonal A/H1N1 influenza virus (n = 19)	Wild-type seasonal A/H1N1 influenza virus (n = 124)
Demographic data		
No. of males (%)	11 (57.9)	73 (58.9)
Mean age ± SD, years	4.33 ± 3.61	3.39 ± 2.58
Previous use of neuraminidase inhibitors, no. (%)	0 (0.0)	0 (0.0)
CT, mean ± SD	25.48 ± 4.44	26.94 ± 4.57
Clinical presentation		
Common cold, No. (%)	4 (21.0)	28 (22.6)
Pharyngitis, No. (%)	5 (26.3)	34 (27.4)
Acute otitis media, No. (%)	3 (15.8)	18 (14.5)
Acute bronchitis, No. (%)	4 (21.0)	26 (21.0)
Pneumonia, No. (%)	1 (5.3)	8 (6.5)
Gastroenteritis, No. (%)	1 (5.3)	6 (4.8)
Fever without source, No. (%)	1 (5.3)	4 (3.2)
Clinical outcome		
Hospitalisation, No. (%)	4 (21.0)	21 (16.9)
Median number of lost school days (range)	6 (1-12)	5 (1-10)
Pharmacological treatment		
Antibiotics, No. (%)	13 (68.4)	88 (70.9)
Antivirals, No. (%)	0 (0.0)	0 (0.0)
Antipyretics, No. (%)	16 (84.2)	99 (79.8)
Socioeconomic impact on households		
Similar disease among family members, No. (%)	10 (52.6)	52 (41.9)
Hospitalisations, No. (%)	1 (5.3)	0 (0.0)
Median number of lost parental working days (range)	3 (1-11)	3 (1-9)
Median number of lost sibling working days, (range)	4 (2-14)	3 (2-15)

CT, cycle thresold; SD, standard deviation. No significant between-group differences.

The findings of this study show that the spread of H275Y-mutated A/H1N1 seasonal influenza virus is a common phenomenon that may be unrelated to the previous use of antivirals, and that the clinical importance and impact on the households of the mutated virus is similar to that of the wild-type in an otherwise healthy pediatric population.

The presence of mutated viruses was marginal in winter 2007-2008, but increased to 100% in the following year even though the use of neuraminidase inhibitors remained minimal in Italy and was not reported in our patients. Due to the lower incidence of A/H1N1 influenza virus in our population during winter 2008-2009 than in the previous year, a small group of children with mutated strains was identified. However, the presence of the H275Y mutation did not seem to modify the pathogenicity of A/H1N1 influenza virus in our otherwise healthy children because all the variables evaluated for the clinical importance and impact on the households of the infections due to mutated or wild-type viruses were comparable. This finding is in line with data coming from national surveillance systems in Europe and the United States [3-6], and suggests that pediatricians do not need to search for this mutation in healthy children with mild to moderate disease.

There have been recent reports concerning the negative evolution of oseltamivir-resistant A/H1N1 influenza [7-9]. However, most of these cases involved subjects with underlying severe chronic diseases, and it is possible that the reduction in host defences due to their underlying condition favoured prolonged viral shedding and the development of severe influenza, as frequently occurs in cases due to wild-type influenza virus. It is also possible that, in cases with a negative evolution, the H275Y mutation may be associated with one or more other genetic mutations. We found only the H275Y mutation in our children who developed mild or moderate influenza, whereas complete sequencing of the neuraminidase gene in adult and elderly patients with severe disease by Goosken *et al*. demonstrated the presence of a second mutation, the T284A substitution [8]. Furthermore, it has been reported that a number of different mutations conferring various degrees of resistance, such as H126N G248R, S247N or S247G, can be found among resistant A/H1N1 influenza strains [5,6], and that the presence of the D344N substitution in neuraminidase is associated with an increase in enzyme activity [15]. All of these data suggest that the risk of severe influenza in subjects with oseltamivir-resistant A/H1N1 virus may be related to the type and number of mutations.

In conclusion, despite its frequency, A/H1N1 influenza virus with a single H275Y mutation is of marginal clinical importance and impact on the households in otherwise healthy children, but further studies are needed to clarify its role in children with severe influenza, as well as the relationships between different mutations and complicated clinical pictures.

## List of abbreviations

(CD): Cycle threshold; (ER): Emergency Room; (PCR): polymerase chain reaction; (RT): reverse-transcription; (SD): standard deviation.

## Competing interests

The authors declare that they have no competing interests.

## Authors' contributions

SE and NP designed the study and co-wrote the manuscript;.GM, CD and AV carried out the real-time PCR; EF, LDD, VC, EB, RG, and CGi visited the patients and collected the swabs; CGa performed the statistical analysis; AL supported in the laboratory assays. All authors read and approved the final manuscript.
